# The Safety of Laser Acupuncture: A Systematic Review

**DOI:** 10.1089/acu.2020.1419

**Published:** 2020-08-13

**Authors:** Juan Yang, Molly J. Mallory, Qinglong Wu, Sara E. Bublitz, Alexander Do, Donglin Xiong, Christina Ying Ying Chen, Peter T. Dorsher, Tony Y. Chon, Brent A. Bauer

**Affiliations:** ^1^ Division of General Internal Medicine, Mayo Clinic, Rochester, MN, USA.; ^2^ Department of Pain Medicine, Shenzhen Nanshan People's Hospital, Shenzhen, Guangdong, China.; ^3^ College of Acupuncture and Rehabilitation, Guangzhou University of Traditional Chinese Medicine, Guangzhou, Guangdong, China.; ^4^ Department of Community Internal Medicine, Mayo Clinic, Rochester, MN, USA.; ^5^ Department of Physical Medicine & Rehabilitation, Mayo Clinic, Jacksonville, FL, USA.

**Keywords:** complementary and alternative medicine, musculoskeletal pain, safety, adverse event, laser acupuncture

## Abstract

**Objective:** Laser acupuncture has become increasingly attractive in clinical practice, especially for patients with needle phobias well as elderly people and children. However, literature concerning the safety of laser acupuncture has been limited. This systematic review synthesizes the current available literature on the safety of laser acupuncture.

**Methods:** Ovid MEDLINE,^®^ Epub Ahead of Print, In-Process & Other Non-Indexed Citations Daily, Ovid Embase, Scopus, and EBM Reviews—Cochrane Central Register of Controlled Trials databases were searched for available randomized controlled trials (RCTs) on laser acupuncture. Safety data were extracted from the included studies. Adverse events (AEs) data were extracted and assessed in terms of severity and causality.

**Results:** Of 737 articles, 21 RCTs were included. The majority of these RCTs reported that laser acupuncture was safe, without AEs; 6 trials reported AEs (including tingling, pain flare-ups, and transient fatigue). All AEs were mild and resolved spontaneously within 24 hours. The causal relationship between AEs and laser acupuncture was felt to be “certain” in 4 studies, “probable” in 1 study, and “possible” in 1 study. AEs were collected and monitored by evaluation methods in 7 trials: 5 with AE questionnaires, 1 with a checklist, and 1 with oral reports.

**Conclusions:** Laser acupuncture appears to be a safe therapy associated with few mild and transient AEs. However, given the heterogeneity of current studies, large, well-designed placebo-controlled RCTs with rigorous evaluation methods are needed to assess the safety of laser acupuncture more completely.

## Introduction

Laser acupuncture is a photonic stimulation of acupoints and areas, initiating therapeutic effects similar to that of needle acupuncture and related therapies together with photobiomodulation.^1^ Laser acupuncture was first developed in China and Russia, and initially applied clinically by Plog on acupoints in 1973–1974.^2,3^ Laser acupuncture is one of recent technological developments (e.g., electroacupuncture, Battlefield Acupuncture) in the practice of acupuncture. Rather than mechanical stimulation produced by traditional needle therapy, acupoints irradiated by the laser acupuncture elicit physiologic effects at the cellular level with sufficient energy.^4^ Because it is a gentle, less-invasive and simple-to-perform nonpharmacologic technique, laser acupuncture has become increasingly attractive for patients with needle phobias, as well as for elderly people and children.^5–7^

Several systematic reviews have shown that laser acupuncture is efficacious for some conditions, such as lateral-elbow tendinopathy, chronic nonspecific low-back pain, musculoskeletal pain, and obesity. However, 1 study did not find laser acupuncture effective for smoking cessation. As this kind of acupuncture becomes more popular, it becomes more important to understand its safety.^4,8–11^

According to a prospective observational study on the safety of traditional acupuncture in 229,230 patients with chronic osteoarthritis pain, the adverse event (AE) risk of acupuncture was 8.6%. While most of the AEs were only minor events, such as bleeding, pain, vertigo, or nausea, 2 patients experienced serious AEs (i.e., pneumothorax), with 1 patient requiring hospital treatment and the other patient needing observation only. Thus, overall, acupuncture was regarded as a relatively safe treatment.^12^ To the current authors' knowledge, the use of laser acupuncture seems to be safe; however, literature concerning the safety of laser acupuncture has been limited. To date, there is no comprehensive review about laser acupuncture's safety. Given the increasing use of this kind of acupuncture as a clinical treatment option, studies on its safety are needed urgently. Therefore, this review systematically collected and synthesized previous published randomized controlled trials (RCTs) regarding the clinical safety evidence of this medical approach.

## Methods

### Search Strategy

**FIG. 1.**
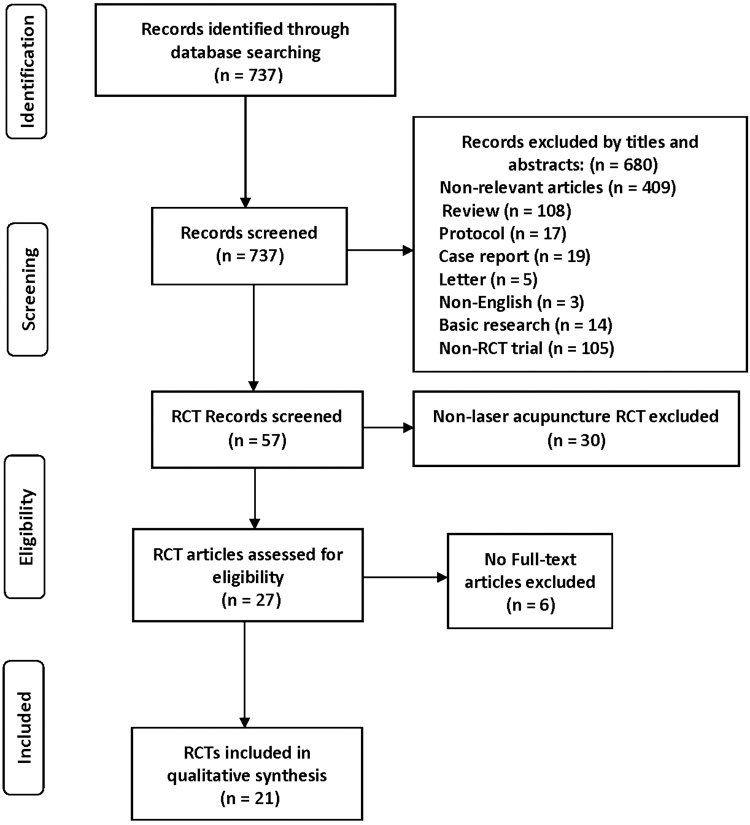
PRISMA flow chart. RCTs, randomized controlled trials.

A comprehensive search was conducted of several databases, from inception to October 1, 2018, limited to English language only, and excluding animal studies. The databases included Ovid MEDLINE,^®^ Epub Ahead of Print, In-Process & Other Non-Indexed Citations and Daily, Ovid Embase, Scopus, and EBM Reviews–Cochrane Central Register of Controlled Trials. The search strategy was designed and conducted by an experienced librarian with input from the study's principal investigator. Controlled vocabulary supplemented with keywords was used to search for studies describing laser acupuncture. Search terms used were *laser therapy* or *laser acupuncture* or *electro-acupuncture* or *acupuncture* or *acupuncture points*. Reference lists of review articles and included relevant studies were hand-searched for additional studies. Commentaries, letters, responses, editorials, and original articles were sought. Randomized controlled trials that mentioned *safe* or *safety* or *adverse event* or *adverse reaction* or *risk* were searched. Given that most articles reported AEs and were indexed poorly, search terms were not combined for *safety* at the cost of sensitivity. Searching was limited to the English language. Figure 1 shows the Preferred Reporting Items for Systematic Reviews and Meta-Analyses (PRISMA) recommended protocol.^13^

### Inclusion/Exclusion Criteria

Trials that met the following criteria were included:

(1) Had original patient data
(2) Involved laser acupuncture treatment for any disease
(3) Reported AEs or had safety reports
(4) Randomized controlled trials (RCTs)
(5) Involved with keeping records of laser acupuncture safety and risks.

Exclusion criteria were as follows:

(1) Reviews
(2) Languages other than English
(3) Studies related to animal and cellular research.

### Data Extraction

Two authors extracted the data independently regarding article author(s), publication year, country, study design, sample size, trial type, disease, interventions, practitioners' information, and detailed information on AEs. Any dispute was resolved by discussion between the 2 authors, and the original study author was contacted for further information by e-mail if needed.

### Data Synthesis

AE data from divergent sources were summarized in a qualitative analysis instead of combining them using meta-analysis. AEs were synthesized from 3 aspects: (1) severity; (2) causality assessment; and (3) evaluation tools.

#### AE severity

This was assessed by two reviewers independently on the basis of the Common Terminology Criteria for Adverse Events (CTCAE) scale V.5.0.^14^ The CTCAE Scale grades 1 through 5 refer to the severity of the AEs. The 5-point grading categories were applied as detailed in Table 1. Any differences were resolved by discussions among the 2 reviewers. If additional data or information was needed, the primary study author was contacted for clarification per Table 1 grading.

**Table 1.** Common Terminology Criteria for AEs

Grades	AEs
1	Mild; asymptomatic or mild symptoms; clinical or diagnostic observations only; intervention not indicated
2	Moderate; minimal, local, or noninvasive intervention indicated; limiting age-appropriate instrumental ADL^a^
3	Severe or medically significant but not immediately life-threatening; hospitalization or prolongation of hospitalization indicated; disabling; limiting self-care ADL^b^
4	Life-threatening consequences; urgent intervention indicated
5	Death related to AE

^a^ Instrumental ADL refers to preparing meals, shopping for groceries or clothes, using the telephone, managing money, etc.

^b^ Self-care ADL refer to bathing, dressing and undressing, feeding self, using the toilet, taking medications, and being not bedridden.

AE, adverse event; ADL, activities of daily living.

#### AE causality

This refers to causal relation between AE and LA treatment, which was assessed by 2 reviewers independently based on the World Health Organisation—Uppsala Monitoring Centre (WHO-UMC) system for standardized case-causality assessment.^15^

#### Tools

 Terminology was modified for use in a device rather than for a therapeutic product, and for clinical trial than a laboratory test. The categories for causality assessment were *certain*, *probable/likely*, *possible*, *unlikely*, *conditional/unclassified*, and *inaccessible/unclassifiable*. Any discrepancy in this procedure was resolved by consensus between the 2 reviewers or by consultation with the article's author.

## Results

### Study Characteristics

For this review, 737 references were identified initially. Of these, 680 irrelevant records were removed based on their titles and abstracts. Of the 57 RCTs, 30 articles were excluded for irrelevance of laser acupuncture safety. After 6 of the 27 non–full-text RCT articles were also excluded, 21 RCTs full-text articles remained for this review.^16–36^ The geographic distribution of these RCTs trials included several countries, with 8 trials that originated in Australia,^19,20,22,27–30,35^ 2 in in the United States,^17,26^ 1 in Germany,^21^ 2 in Indonesia,^24,34^ 1 in Iran,^18^ 1 in Denmark,^31^ 1 in Egypt,^25^ 1 in Scotland,^16^ 1 in Austria,^32^ 1 in China,^23^ 1 in India,^36^ and 1 in Brazil.^33^ See Table 2A.All the included trials were ethically approved and informed consent was obtained from all patients and parents (when the subjects were children).

**Table 2A.** Characteristics of Included RCTs: Trial Types, Lasers Used, and Efficacy

1st Author	Year	Country	N	Condition	Laser Type	Wave type	Wavelength	Output	Density	Dose	Efficacy
Basford^17^	1987	USA	81	Thumb osteoarthritis	Low-energy helium neon laser	Continuous	632.8 nm	0.9 mW	—	—	Negative
Naeser^26^	2002	USA	11	Carpal tunnel syndrome pain	Low level laser (Red-beam laser/infrared laser)	Continuous	632.8 nm or 904 nm	15 mW & 9.4 W	225 J/cm^2^ or 1.81–0.04 J/cm^2^	7 J/point	Positive
Quah-Smith^30^	2005	Australia	30	Mild-to-moderate depression	Low level infrared laser	—	—	100 mW	—	0.5 J/point	Positive
Ebneshahidi^18^	2005	Iran	50	Chronic tension-type headache	Infrared laser (Ga-As-Al)	Continuous	830 nm	39 mW	13J/cm^2^	1.3 J/point	Positive
Stockert^35^	2007	Australia	17	Pediatric asthma	Diodes soft laser	—	670 nm	10 mW	—	—	Positive
Gottschling^21^	2008	Germany	43	Pediatric headache	Infrared laser	Continuous	830 nm	30 mW	3.8 W/cm^2^	0.9 J/point	Positive
Glazov^19^	2009	Australia	100	Nonspecific low-back pain	Infrared laser(Ga-As-Al)	Continuous	830 nm	10 mW	0.05 W/cm^2^	0.2 J/point	Negative
Radvanska^31^	2011	Denmark	31	Monosymptomatic nocturnal enuresis	Laser acupuncture	—	670 nm	10 mV	—	—	Negative
Moustafa^25^	2013	Egypt	40	Pediatric allergic rhinitis	Low-level infrared laser	Frequent	905 nm	30 W	10,000-Hz frequency	0.12 J/point	Positive
Quah-Smith^29^	2013	Australia	16	Differential brain effects	Moxla prototype fibrotic infrared light laser	Continuous	808 nm	20 mW	—	—	Negative
Quah-Smith^28^	2013	Australia	20	Major depression	Low-level infrared laser	Continuous	808 nm	25 mW	—	0.5 J/point	Negative
Quah-Smith^27^	2013	Australia	47	Major depression	Low-level infrared laser	Continuous	808 nm	100 mW	—	1 J/point	Positive
Hinman^22^	2014	Australia	282	Chronic knee pain	Low-level red light laser	—	—	10mW	—	0.2 J/point	Negative
Glazov^20^	2014	Australia	144	Nonspecific chronic low-back pain	Low-dose infrared laser	Continuous	830 nm	20 mW	0.1 W/cm^2^	0, 0.2, or 0.8 J/point	Negative
Al Rashoud^16^	2014	Scotland	49	Knee osteoarthritis	Low-dose infrared laser	—	830 nm	30-mW	4J/cm^2^	1.2 J/point	Positive
Raith^32^	2015	Austria	28	Neonatal abstinence syndrome	Infrared laser	Continuous	675 nm	10 mW	17 or 34 J/cm^2^	0.3 or 0.6 J/point	Positive
Hung^23^	2016	China	66	Postpartum weight	Infrared laser	—	810 nm	150 mW	5W/cm^2^	0.375 J/point	Negative
Srilestari^34^	2017	Indonesia	36	Diabetic foot ulcer	Low-level diode laser(Red light laser)	—	630 nm	100 mW	—	4 J/point	Positive
Mihardja^24^	2017	Indonesia	29	Plasma levels of β-endorphin in healthy subjects	Infrared laser	Continuous	785 nm	50 mW	20 or 35–40 mW/cm^2^	4 J/point	Positive
Goel^36^	2017	India	40	Pediatric gag reflex	Low-level laser (diode laser)	Continuous	940 nm	0.5 mW	—	4 J/point	Positive
Sampaio-Filho^33^	2018	Brazil	84	Postoperative pain in third molar surgery	Low-level laser (red diode laser )	—	660 nm (± 10 nm)	100 mW	35.4 mW/cm^2^	1 J/point	Positive

RCTs, randomized controlled trials; AEs, adverse events; Ga-As-Al, gallium-arsenide-aluminium.

**Table 2B.** Characteristics of Included RCTs: Practitioners, Safety Measures, and Patients' Responses

1st Author	Year	Provider	Full contact	Protective goggles	Other	AEs definition	Evaluation	AEs report	Severity	Causality
Basford^17^	1987	—	—	Y		Good or bad pain	Question & normal scale	Tingling	Mild	Certain
Naeser^26^	2002	Acupuncturist	Y	—		—	—	No	—	—
Quah-Smith^30^	2005	Acupuncturist	Y	—		Fatigue, insomnia, dry mouth & headache	AEs Questionnaire	Transient fatigue, insomnia, dry mouth & headache	Mild	Certain
Ebneshahidi^18^	2005	Acupuncturist	Y	—		—	—	No	—	—
Stockert^35^	2007	Acupuncturist	—	Y	Other	—	—	No	—	—
Gottschling^21^	2008	Acupuncturist	—	Y		Eye irradiation	—	No	—	—
Glazov^19^	2009	Acupuncturist	Y	—		Pain exacerbations	Oral report	Pain flare-up	Mild	Unlikely
Radvanska^31^	2011	Investigator supervised by an acupuncturist	—	—		—	—	No	—	—
Moustafa^25^	2013	—	—	Y		—	—	No	—	—
Quah-Smith^29^	2013	Acupuncturist	—	—		Dizziness, aches, transient fatigue, prolonged fatigue, vagueness and nausea	6-item scale	Tingling, transient tiredness, dizziness, vagueness & nausea	Mild	Certain
Quah-Smith^28^	2013	Acupuncturist	—	—		—	—	No	—	—
Quah-Smith^27^	2013	Acupuncturist	Y	Y		Dizziness, aches, transient fatigue, prolonged fatigue, vagueness & nausea	6-item scale	Minimal transient fatigue	Mild	Possible
Hinman^22^	2014	Acupuncturist	—	—		“No pain” & “Worst pain possible”	NRS questionnaire	No	—	—
Glazov^20^	2014	Experienced general-practitioner therapists	Y	—		“Worst pain possible”	Checklist	Pain flare–up & other symptoms	Mild	Certain
Al Rashoud^16^	2014	Trained physiotherapist	Y	Y	Device routine check	Eye irradiation	—	No	—	—
Raith^32^	2015	Acupuncturist	Y	Y	Device management	Eye irradiation	—	No	—	—
Hung^23^	2016	Acupuncturist	-	—		—	—	No	—	—
Srilestari^34^	2017	—	-	Y		—	—	No	—	—
Mihardja^24^	2017	—	-	—		Pain	—	No	—	—
Goel^36^	2017	—				—	—	No	—	—
Sampaio-Filho^33^	2018	Acupuncturist	—	—		—	—	No	—	—

RCTs, randomized controlled trials; AEs, adverse events; NRS, numeric rating scale.

**FIG. 2.**
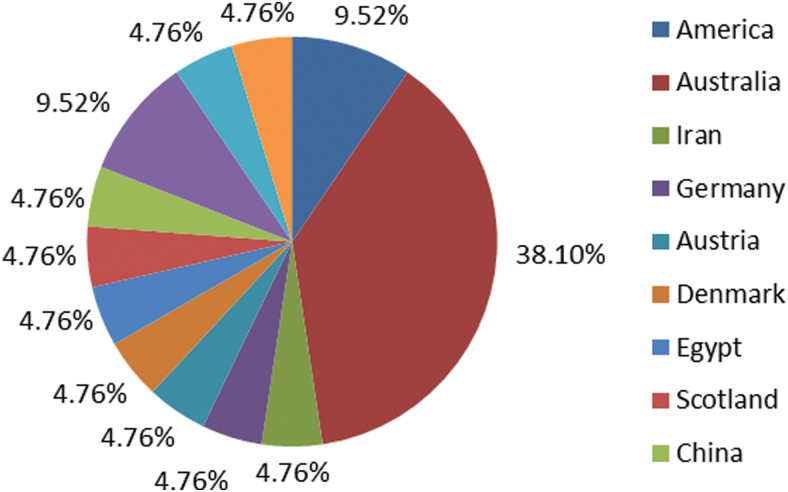
Geographic distributions of randomized controlled trials related to laser acupuncture safety. Color images are available online.

A large proportion of AEs in the literature reports originated from European and North American countries. (Fig. 2) The majority of RCTs^16,18,21–26,28,31–36^ reported that laser therapy was safe without AEs. AEs varied substantially among 6 studies, mainly including: tingling^17,29^; pain flare-up^19,20^; fatigue^27,30^; insomnia, dry mouth, and headache^30^; and transient tiredness and dizziness, vagueness, and nausea.^29^

The treatment in 13 studies was administered by a licensed acupuncturist,^18,19,21–23,26–30,32,33,35^ 1 study by an investigator supervised by a certified acupuncturist, 1 by experienced general practitioner therapists,^20^ 1 by a trained physiotherapist,^16^ and 5 without mention of providers.^17,24,25,34,36^ See Table 2B. Among all the included trials, 62% reported positive outcomes of laser acupuncture's clinical efficacy.^16,18,21,24–27,30,32–36^ For safety, safeguard measurements were given to medical providers; patients were instructed to wear goggles to shield their eyes^16,17,21,25,27,32,34,35^; and the laser probe was fully in contact with each participant's skin surface^16,18–20,26,27,30,32^ to deliver active laser radiation. Characteristics of included RCTs can be seen in Table 2.

### Adverse Events

#### Tingling

Two trials reported tingling with laser acupuncture. As a noninvasive intervention applied to the skin, laser acupuncture produced no sensation and did not burn the skin,^26^ but there was tingling reported at the site of laser acupuncture manipulation.

In 1 trial evaluating the efficacy of low-energy helium-neon (HeNe) laser treatment for thumb osteoarthritis (OA), 81 patients were randomized into a treatment group (*n* = 47, 0.9 mW continuous wave (HeNe laser) and a control group (*n* = 34, sham treatment). The affected thumbs of both groups were “irradiated” with a laser beam for 15 seconds, thrice weekly for 3 weeks. One participant in the treatment group reported transient tingling on the distal superficial branch of the radial nerve; this sensation disappeared spontaneously within 24-hours. The researchers concluded that HeNe laser irradiation at 0.9 mW was safe.^17^

In the other study, 16 healthy volunteers were randomized to receive low-intensity laser acupuncture on one side to LR 8 and needle acupuncture on the other side to LR 8. Stimulation was in alternating rest/active phases, and brain patterns were recorded by a 3T Philips Intera magnetic resonance imaging scanner. Both interventions at LR 8 produced different brain patterns. All subjects reported feeling the touch of the laser probe but only a few patients felt any sensation produced by the laser beam itself, one feeling warmth, 3 feeling tingling, and 1 reporting both sensations.^29^

#### Pain flare-up

Two trials reported pain flare-ups with laser acupuncture. Glazov et al^19,20^ mentioned the AE of pain flare-ups related to laser acupuncture in 2 trials of patients with chronic low-back pain.

In 1 double-blinded, 2-group parallel RCT the efficacy of a gallium–arsenide–aluminum (Ga-Al-As) laser-diode laser on pain and disability was investigated in patients with chronic nonspecific low-back pain, compared with a sham control. Results showed that 59% participants had minor pain flare-ups and 6% had major flare-ups, but there was no statistically significant difference between the 2 groups.^19^

In the other trial, 144 patients were randomized to sham (0 J/point), low-dose (0.2 J/point), or high dose (0.8 J/point) groups for 8 once-weekly treatments. No pain- or disability-relieving difference was found among the groups. About 28% of treatments were recorded as followed by flare-ups of low-back pain within 1 week after treatment and there were no obvious differences in the flare-up frequencies.^20^

#### Transient fatigue

Two trials reported transient fatigue with laser acupuncture. Quah-Smith and her colleagues^27,30^ reported transient fatigue after low-level laser acupuncture treatment in patients with mild-to-moderate depression.

In 1 trial, 30 participants were randomized to either active or inactive laser treatment trial groups. In the active treatment group, 0.5-J laser treatment was delivered to each of 6–8 acupuncture sites per visit, twice weekly for 4 weeks, then once weekly for another 4 weeks. About 29% of the active group reported AEs, and 17% of the inactive group reported AEs. Fatigue was the most-common complaint in the former group (60%).^30^

In the other double-blinded RCT, 47 participants, ages 18–50, were randomized to 2 groups who received laser acupuncture or placebo laser at acupoints CV 14, HT 7, LR 14, LR 8, and KI 3, twice weekly for 4 weeks and once per week for a further 4 weeks—12 sessions in total. After the 12 sessions were completed, minimal transient fatigue was noted in the laser acupuncture group; while transient fatigue, aches, days of fatigue, and vagueness were noted in the placebo group. No significant AE differences between the 2 groups were seen.^27^

All of the fatigue in both trials was mild and transient, which disappeared without special treatment within 24 hours after the acupuncture.

#### Other *adverse events*

Some other adverse events related to laser acupuncture—such as insomnia, dry mouth, and headache, transient tiredness and dizziness, and vagueness and nausea were also reporded.^29,30^ They were mild and transit and resolved spontaneously within 1 day post treatment.

### Severity and Causality Assessment of AEs Associated with Laser Acupuncture

AEs in the 21 RCT reports were examined in 1244 participants regarding causality. Among the trials, only 4 were evaluated as certain causality; the rest included 1 as probable, 1 as possible, and 0 were unlikely, conditional/unclassified, or inaccessible/unclassifiable cases. In addition, all of the AEs were evaluated as mild in severity assessment. Severe AEs or deaths related to AEs were all regarded unlikely to have been caused by laser acupuncture treatment.

Among the 21 analyzed trials, just 7 (33%) provided explicit information on the methods used to monitor or collect AE data. Accounting for the AE evaluation methods of the 7 trials included: 5 collected with AE questionnaires^17,22,27,29,30^; 1 with a checklist^20^; and 1 with oral reporting.^19^ In 1987, during a study of a low-energy He laser for thumb OA, each participant was asked if there was a “good” or “bad” effects since the last treatment, at the first, third, sixth, and last treatment.^17^ Questions were also asked about pain, stiffness, activity, and medication usage, and the responses were quantified with ordinal scales ranging from 0 to 4 or 5. After completion of treatment, questions regarding late-occurring benefits or AEs were asked in telephone follow-ups.^17^

Quah-Smith et al.^30^ sent an AE questionnaire to participants after they completed their final treatments in 2005 and found that 29% participants of the active laser group and 17% of the placebo group reported AEs.

In two trials in 2013, after 12 sessions of treatment, all of the participants were asked to complete a scale with a score range from 0 to 6 to assess any AEs (aches, dizziness, nausea, prolonged fatigue, transient fatigue, and vagueness).^27,29^ In the trial evaluating the efficacy of laser acupuncture for chronic nonspecific low back pain, AEs post each treatment were documented on a checklist regarding with pain flare-ups occurrence and other symptoms.^20^ In another trial to observe AEs and pain exacerbations during treatment, the study therapist and assessors were required to not comment during the treatment, and follow-up surveys were assessed orally. Participants were asked how they were over the previous week; no side-effects were documented apart from pain variations.^19^

## Discussion

In this review, all the identified AEs were mild and transient, suggesting that laser acupuncture appears to be safe. Despite these favorable findings, laser acupuncture might still be associated with some significant risks in certain circumstances. The laser device emits visible and/or invisible laser beams, which can cause irradiation to the eyes. *High* doses of *radiation* therapy are more likely to stimulate target tissues and cause AEs.^18^ According to European Norm (EN 60825-1), low-level lasers were classified in the category of 3R category, which was equivalent to the old classification, 3B: radiation could potentially cause serious damage to the eyes, therefore eye protection is critical and required by law.^37^ Eye irradiation of laser acupuncture treatment was mentioned as having the potential to cause common and severe AEs, such as possible damage to the conjunctiva and retina etc.,^16,21,32^ while these AEs did not occur in any included trial of our review. This might be attributed to the more-routine use of proper protective measures. All the studies were reviewed and approved by the research ethics committees of the institutions where the trials occurred.

Participants, parents, or persons having the custody of patients who are considering receiving laser acupuncture must be given accurate and detailed information about the research intervention and possibility of AEs. Safeguard measurements for laser acupuncture in the clinical setting were obviously indicated in this current review, such as before the laser was switched on, the robe of the laser device should have been perpendicularly in contact with the skin and all the people in the room had been instructed to wear protective goggles against radiation that could damage their eyes. Newborns or infants who were receiving laser acupuncture interventions had eye protectors to cover their eyes.^32^ The effectiveness of protections, as required for Class 3B lasers, and application of the low-level laser were tested and approved by the administration departments of the institutions that carried out the studies,^32^ and the research laser devices were routinely checked to ensure proper functioning.^16^

These guideline and precautions should be addressed by other practitioners in training; regulating the operations, therapeutic dose, and protections can reduce the high risks of irradiation.

The evidence for the safety of laser acupuncture is not conclusive, but this can be improved in future trials by simple measures, such as utilizing uniform definitions of AEs for all studies. Explicitly including assessment of AEs in future studies, objectively recorded and analyzed, is also a critical step in the development of a more-robust and scientifically valid safety assessment of laser acupuncture. The specific AE questionnaires and symptom checklists as used in several of the reviewed studies may be an excellent resource for data monitoring and collecting.

### Strengths and Limitations of This Review

To the current authors' knowledge, this is the first systematic review to evaluate the safety of laser acupuncture therapy. Some limitations of this review should be noted. For example, the electronic search was initially conducted with the limitation to publications relevant to safety only. This may have led to the potential exclusion of some important information on the clinical efficacy of laser acupuncture. Furthermore, due to the diversity of laser parameters (such as wave types, lengths, outputs, and densities), it would be premature to try to draw conclusions about minimal effective dosages. Therefore, all the available laser parameters from the reviewed studies were included in this review, with the hope of forming a foundation for future systematic reviews of laser acupuncture efficacy. In addition, most studies did not provide appropriate information regarding AE incidence reporting. In this review, AEs were reported by only 32% of the included studies, and, due to unclear prescriptions in some of the original publications—such as vague AEs, number of sessions occurred, unclear AE case numbers, etc.—it might be difficult to draw any meaningful conclusions about AE occurrence-incidence reports. Future studies, performed rigorously—with specific emphasis on identifying and recording AEs—are needed.

## Conclusions

Laser acupuncture, performed within standardized guidelines and in compliance with all safety precautions, seems to be a safe and well-tolerated therapy that is associated with few mild and transient AEs. Large, well-designed placebo-controlled RCTs with rigorous evaluation methods are needed in the future.
